# Early weaning in idiopathic scoliosis

**DOI:** 10.1186/s13013-015-0059-2

**Published:** 2015-11-19

**Authors:** Harald Steen, Johan Emil Lange, Jens Ivar Brox

**Affiliations:** Biomechanics Laboratory, Department of Orthopaedics, Oslo University Hospital, PB 4959, Nydalen, Oslo N-0424 Norway; Section of Spine Surgery, Department of Orthopaedics, Oslo University Hospital, PB 4959, Nydalen, Oslo N-0424 Norway; Department of Physical Medicine and Rehabilitation, Oslo University Hospital, PB 4959, Nydalen, Oslo N-0424 Norway

**Keywords:** Idiopathic scoliosis, Scoliosis treatment, Brace weaning, Curve progression

## Abstract

**Background:**

Many years of bracing represent a burden to the patients. Early weaning may be the result of poor compliance, but may also be planned in patients with a long expected treatment time and a reduced stable primary curve during bracing. The aim of the present cohort study was to compare curve size, health related quality of life and surgical rates at long-term follow-up after ordinary bracing, planned and unplanned early weaning.

**Methods:**

Three hundred eighty-one patients (353 girls/28 boys) with late-onset juvenile (*n* = 30) and adolescent (*n* = 351) idiopathic scoliosis and a mean primary major curve of 33.1 (range 20–57)° were treated with Boston brace and followed prospectively.

**Results:**

Ordinary brace treatment was completed in 290 (76 %) patients, planned early weaning at bone age <14 years in 59 (16 %), and unplanned early weaning in 32 (8 %), while 14 (5 %), 1 (2 %), and 12 (38 %) had surgery, respectively. Forty-eight (81 %) of the patients had a primary curve ≤ 25° at planned early weaning. Six-teen (27 %) of those who had planned early weaning, resumed bracing after a mean time of 2.0 years. The mean curve size at long-term follow-up in average 23.4 years after weaning, was smaller (*p* < 0.001) in patients with planned early weaning (25.1°) compared with ordinary bracing (34.0°) and unplanned early weaning (34.8°). Patient satisfaction and self-image at long-term was better in the planned early weaning group (*p* < 0.05), but differences were small.

**Conclusion:**

The benefit of planned early weaning was the shortened bracing time and good clinical results. This procedure may be attempted if curve reduction is stable over time and the primary curve is 25° or less in patients with several years of expected bracing. The patients should be monitored carefully and regularly at 4-6 months intervals until maturity, and a new brace should be prepared if the curve increases significantly.

## Background

A Cochrane review concluded that there is very low quality evidence in favour of using braces for adolescent idiopathic scoliosis [1]. Later a recently published randomised controlled trial (BRAIST study) found that brace treatment is effective in reducing the number of patients who progress to 50° [2]. The rate of success was 72 % after bracing compared with 48 % after observation. A number of patients used the brace less than planned and the benefit of bracing increased with longer hours of brace wear. Results are in agreement with previously published cohort studies with >19 years follow-up [3–6]. We reported that the surgical rate increased with poor compliance and aborted brace wear [7]. In a subgroup of these patients early weaning was planned and we have not previously reported the results of this strategy. The aim of the present study was therefore to evaluate progression of the scoliotic curve and the surgical rate in patients with unplanned early weaning (aborted brace wear), planned early weaning and ordinary brace wear until maturity.

## Methods

Three hundred eighty-one patients (353 girls and 28 boys) with Boston brace treatment for idiopathic scoliosis were included and followed prospectively [7]. The reason for bracing was adolescent idiopathic scoliosis (AIS) discovered at 10 years of age or older [8] in 351 patients, and late-onset juvenile idiopathic scoliosis at 7 to 9 years in 30 patients. The mean primary major curve measured 33.1 (range 20–57)°. The indication for bracing was a major scoliotic curve ≥ 20° with an observed progression >5° after 4 months and Risser sign <3. Patients had follow-ups with clinical and radiological examination at 4-6 months intervals throughout the brace treatment period. Wearing of the brace was assessed by one of the authors (orthopaedic scoliosis surgeon JEL) and reported as: ordinary as prescribed, irregular, or aborted. Patients were recommended to use the brace for 23 h daily. Wearing of the brace <20 h daily was described as irregular. Initially bracing was planned until maturity (Risser 5 in boys or Risser 4 or two years after menarche in girls). Because some of the patients started bracing at a young age with a long time treatment perspective, the original plan was changed and the idea of planned early weaning to shorten the years of brace wear was concepted and realized 35 years ago by the senior author (JEL). He started early weaning in patients before reaching puberty after 2–3 years or more of brace treatment. These patients demonstrated a good effect of the brace with a stable reduction of the primary curve to 25° or less at repeated measurements. Later, influenced by the positive results associated with this new regimen, he also included a few patients with less than 2 years of brace wear. All had skeletal age <14 and Risser 3 or lower at planned early weaning. The weaning procedure was performed during a period of 2–3 months by 2 h less bracing time every week and the curve was controlled after 4 months. Patients with early weaning were followed at regular intervals of 4–6 months until skeletal maturity. After weaning patients with ordinary brace treatment time had follow-ups at 6, 12, and 24 months. After several years a long time follow-up was accomplished in all the patients.

### Radiology

Prior to bracing standing radiographs were taken in the front and lateral projections. In addition non-weight bearing (flexibility) frontal radiographs were exposed in the prone position, and after brace fit standing (redressment) radiographs with the brace on were taken. Radiological measurements were performed by an orthopedic surgeon (JEL) and controlled by an experienced radiologist. Both used the Cobb method manually. Digital measurements were used at long-term follow-up. The intra-observer error for the Cobb angle was about 3° in a relatively recent study using manual and digital measurements, and <5° in a previous study [9, 10]. In the present study the measurement error was within these limits as evaluated by the reproducibility of radiographic readings of repeated measurements of all radiographs from 10 patients at regular intervals. In patients with double thoracolumbar curves the largest curve prior to bracing was defined as the major curve.

### Surgery

Surgery was recommended in patients with curve progression to >45° during bracing and at weaning. At later follow-up patients with major curves <50° were not recommended surgery. Information about surgery was recorded in a standardised form and obtained from a long-term questionnaire and checked in the medical journals.

### Questionnaires

At long-term follow-up, a standardised questionnaire was filled in by the patients. The questionnaire comprised validated measures of pain, disability, quality of life and work, comorbidity, surgery, and questions about demographics.

A validated Norwegian version of the Scoliosis Research Society 22 questionnaire (SRS-22) was used for evaluation of health related quality of life [11]. The SRS-22 covers four domains (function/activity, pain, self-perceived image, mental health) each with 5 questions, and one domain (satisfaction with treatment) with 2 questions. Each item has 5 verbal response alternatives ranging from 1 (worst) to 5 (best). Results are expressed as the mean (total sum of the domain divided by the number of items answered) for each domain.

Patients rated their overall function by the Global Back Disability Question [12]. This is a single question designed to measure the patients’ overall rating of their back disability today. There were five response alternatives: “excellent, none or unimportant complaints", “good, occasionally bothered by back pain", “fair, some back pain and limited function", “poor, unchanged, considerable complaints and severe disability", and “miserable, worse, not self-reliant in activities of daily living”.

A Norwegian version of the original Oswestry Disability Index (version 1.0) was used to evaluate back-specific disability [12]. The sum of 10 questions is calculated and presented as a percentage, wherein 0 % represents no pain and disability, and 100 % represents the worst pain and disability.

The General Function Score was used to measure disability in activities of daily living [13]. Patients answered nine questions using one of three alternatives: “can perform”, “can perform with difficulty due to back complaints”, and “cannot perform due to back complaints”. The score was presented as a percentage wherein 100 % represents maximum disability.

EuroQol is a generic (non-disease specific) questionnaire for measurement of health related quality of life and includes five items regarding quality of daily life, covering the domains of mobility, self-care, usual activities, pain and discomfort, and anxiety and depression (EQ-5D) and a visual analogue score (EQ-VAS) for assessment of overall current health [14, 15].

### Statistical analysis

Results are presented as means and range or standard deviation, or as percentages. The normal distribution of baseline, follow-up data, and differences were checked by histograms and by the Shapiro-Wilk test. In sample cases of non-normal distribution non-parametric methods were used. A General Linear Model One-way analysis of variance was applied to test differences between the 3 subgroups in continuous variables at baseline, weaning, and follow-up: 1) patients with unplanned early weaning, 2) planned early weaning, and 3) ordinary brace wear until maturity. In a previous study we classified irregular wear and unplanned early weaning as poor compliance [7]. In the present study we classified patients with irregular wear as ordinary bracing unless bracing was aborted. With the assumption of unequal variances in unequally sized groups, Dunnett’s T3 was used for post hoc multiple comparisons. Chi-square analyses were applied for testing of categorical variables and Kendall’s Tau-b measure of association for multiple nominal variables. Statistical evaluation was performed by use of the Statistical Analysis System (SAS version 9.2; Cary, NC) and by SPSS software, version 21.0 (SPSS Inc., Chicago).

### Consent

The committee for medical research ethics in the health Region South-East in Norway and the institutional review board (hospital’s patient ombudsman) approved the study (REK 2010-3677). Written informed consent was obtained from the participating patients.

## Results

Ordinary brace treatment was completed in 290 (76 %), while 14 (5 %) of these patients were operated due to curve progression in spite of brace treatment. Early weaning was planned in 59 patients, 16 (27 %) of these started a new period of brace treatment and 1 (2 %) was operated. Early weaning was unplanned in 32 patients, mainly due to pain, psychological distress or skin problems. Among these patients, bracing was resumed in 6 (19 %), and 12 (38 %) were operated. The surgical rate was significantly different in patients with ordinary bracing, planned and unplanned early weaning (*p* < 0.005; Kendall’s Tau-b).

### Major curves at baseline

**Fig. 1:**
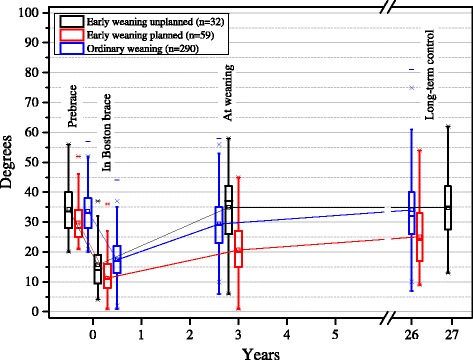
Overall primary scoliotic curve development in 381 Boston braced patients with late-onset juvenile or adolescent idiopathic scoliosis. Patients with resumed bracing and operated patients included

At start of brace treatment chronological age and skeletal age assessments were significantly (*p* < 0.001) lower in the early weaning patients compared to the patients with ordinary bracing (Table 1). In general bone age was lower than the calendar age, and in patients with planned early weaning the average difference (1.0 year) between skeletal age and chronological age was significantly (*p* < 0.001) larger than in the group of patients with ordinary bracing. The primary scoliotic curve development is presented in Fig. 1. The average major curve standing without brace at the start of treatment was 33.1° and significantly lower in the patients with planned early weaning (29.8°) compared to those with unplanned early weaning (34.3°; *p* < 0.005) and those with ordinary bracing (33.6°; *p* < 0.001). Also both the average major curve prone without brace and standing in brace were significantly lower (19.1° and 11.3°) in the planned early weaning group compared to the unplanned early weaning (24.3° and 15.7°) and ordinary bracing (23.7° and 17.4°) groups, respectively. Correspondingly, the major curve’s mean percentage flexibility (36.1 %) and redressment (62.9 %) in the planned early weaning group were significantly larger relative to the other groups.

**Table 1:** Baseline characteristics in 381 Boston braced patients

Characteristic	A. Unplanned early weaning	B. Planned early weaning	C. Ordinary bracing
	*n* = 32	*n* = 59	*n* = 290
Age at start brace treatment (years)	11.5 (7.7–15.5)	11.4 (7.5–16.0)	13.7 (6.9–17.1)^a^
Bone age at start brace treatment (years)	10.8 (7–14)	10.4 (5–13)	13.2 (7–16)^a^
Bone – Chronological age difference at start brace treatment (years)	−0.8 (−2.7–0.8)	−1.0 (−4.0–2.0)^b^	−0.5 (−3.2–2.1)
Age at menarche (years)	13.6 (11–16)	13.7 (11–16)	13.4 (7–19)
	(*n* = 25)	(*n* = 51)	(*n* = 262)
Major curve standing without brace at start of treatment (°)	34.3 (20–56)	29.8 (21–52)^c^	33.6 (20–57)
Major curve prone without brace at start of treatment (°)	24.3 (8–41)	19.1 (7–42)^d^	23.7 (9–45)
Major curve standing in brace (°) at start of treatment	15.7 (4–37)	11.3 (1–37)^c^	17.4 (1–44)
Major curve flexibility (°)	10.0 (1–22)	10.7 (2–23)	9.9 (−2–24)
Major curve flexibility (%)	29.1 (5–73)	36.1 (7–70)^e^	29.6 (−5–69)
Major curve redressment (°)	18.7 (1–46)	18.6 (8–33)	16.2 (3–35)^f^
Major curve redressment (%)	53.7 (4–86)	62.9 (25–97)^e^	48.9 (7–96)

Values are means (range)

^a^Different from A and B (*p* < 0.001)

^b^Different from C (*p* < 0.001)

^c^Different from A (*p* < 0.005) and C (*p* < 0.001)

^d^Different from A (*p* < 0.001) and C (*p* < 0.001)

^e^Different from A (*p* < 0.05) and C (*p* < 0.001)

^f^Different from A (*p* < 0.05) and B (*p* < 0.01)

### Major curves at weaning and long - term follow-up

At weaning both the chronological age and bone age was highest in the ordinary bracing group (Table 2). The average difference between skeletal age and chronological age at weaning was larger in the planned early weaning group versus the ordinary bracing group.

**Table 2:** Results at weaning and last follow-up control in 381 Boston braced patients

Characteristic	A. Unplanned early weaning	B. Planned early weaning	C. Ordinary bracing
	*n* = 32	*n* = 59	*n* = 290
Age at weaning (years)	14.3 (9.6–16.6)	14.9 (11.0–18.9)^a^	16.3 (12.4–19.8)^b^
Bone age at weaning (years)	13.6 (9–15)	13.9 (11–16)	15.8 (11–18)^b^
Bone – Chronological age difference at stop brace treatment (years)	−0.7 (−3.6–2.1)	−1.0 (−4.0–2.0)^c^	−0.4 (−3.2–2.5)
Time in brace at weaning (months)	31.9 (6–82)	36.4 (12–71)^d^	31.1 (6–113)
Number of patients in brace time subgroups (months) <24 / ≥24 and <36 / ≥36 and <48/≥48	16/7/1/8	16/18/12/13	91/117/56/26
Major curve standing at weaning (°)	35.0 (6–58)	20.6 (1–45)^e^	29.3 (6–58)^f^
Time to last follow-up after weaning (years)	24.2 (2.7–30.4)	23.1 (15.0–30.7)	23.4 (2.4–32.1)
Major curve standing at last follow-up (°)	34.8 (13–62)	25.1 (9–54)^e^	34.0 (7–81)
Major curve difference at last follow-up relative to start bracing (°)	0.5 (−23–21)	−4.7 (−28–23)^a^	0.5 (−22–51)
Number (%) operated^g^	12 (38)	1 (2)	14 (5)

Patients with resumed bracing and operated patients included. Values are means (range)

^a^Different from A (*p* < 0.05) and C (*p* < 0.001)

^b^Different from A and B (*p* < 0.001)

^c^Different from C (*p* < 0.001)

^d^Different from C (*p* < 0.05)

^e^Different from A (*p* < 0.001) and C (*p* = 0.001)

^f^Different from A (*p* = 0.005) and B (*p* < 0.001)

^g^Kendall’s Tau-b −0.1929 (−0.3223 to −0.0635); *p* < 0.005

The patients who resumed bracing saved a mean time of 2.0 (0.4–3.9) years in the brace, while the average total time bracing was 36.4 months and significantly (*p* < 0.05) longer in the planned early weaning group compared to the patients with ordinary bracing (31.1 months).

At weaning the standing major curve without brace in the patients with planned early weaning was in average 20.6° compared with 29.3° in the ordinary bracing group, and 35.0° in the patients with unplanned early weaning.

At the last long-term follow-up control the final average standing major curve with operated patients included was in average 25.1° and signficantly less in the planned early weaning group compared to the unplanned (34.8°) and ordinary bracing (34.0°) groups, respectively. The change in the major curve from baseline was in average a significant reduction of 4.7 ° in the planned early weaning group, while it was 0.5° increased in the two other groups (Table 2).

### Planned early weaning

Fortyeight (81 %) of the patients had a primary curve ≤ 25° at weaning.

Sixteen (27 %) of the patients resumed bracing. All these patients had initially a curve reduction >5°; the mean reduction was 14.8° (SD 6.1). The curve increased in average 15.4° (SD 3.7) during a mean time of 2 years before bracing was resumed.

### Questionnaires

**Fig. 2:**
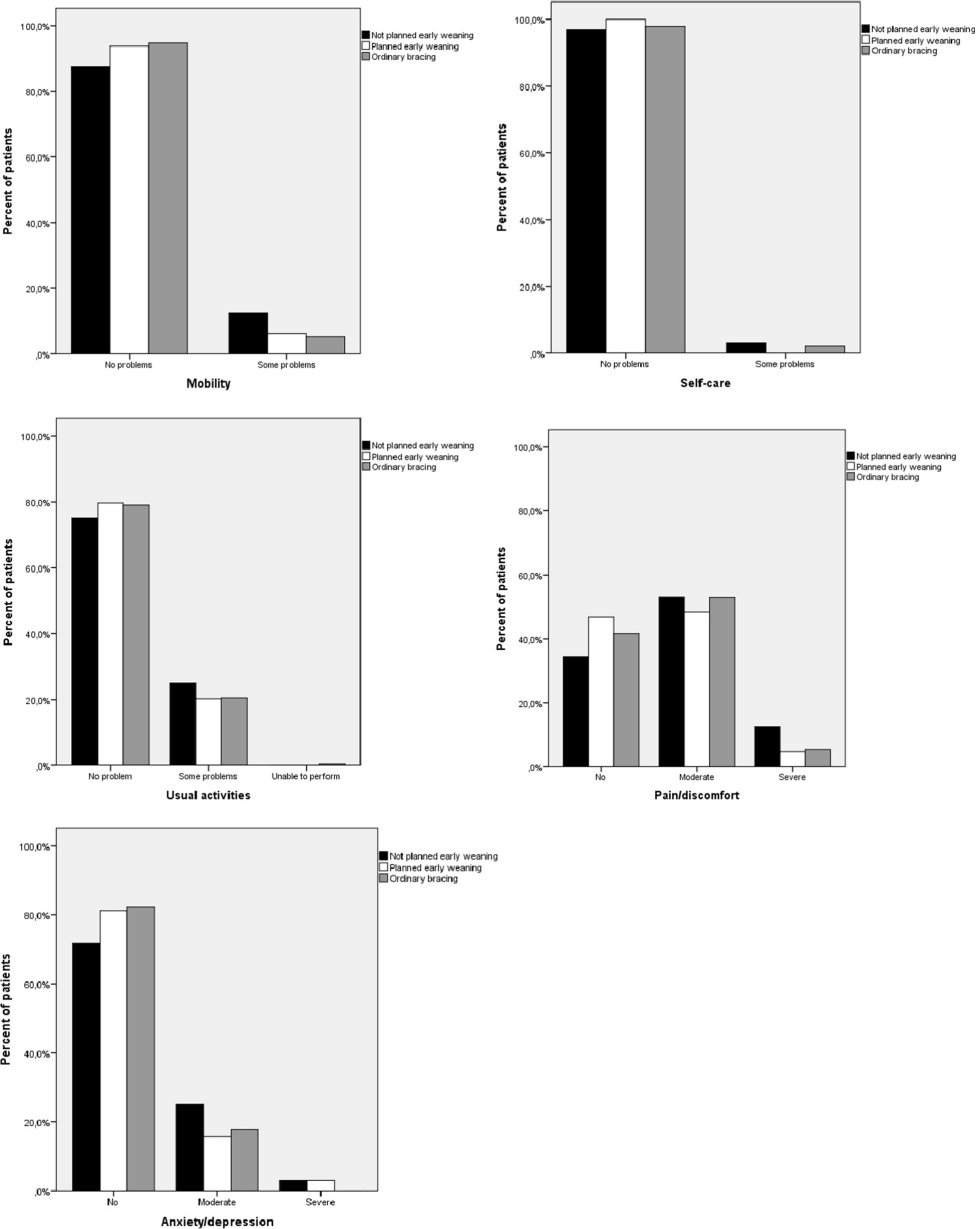
EQ-5D results in 381 Boston braced patients with late-onset juvenile or adolescent idiopathic scoliosis at mean follow-up of 23.4 years. Percentages are given

Sociodemographics and quality of life at long-term are presented in Tables 3 and 4 and in Fig. 2. There were in general small differences between groups. Scores suggest that quality of life for most patients are within the average population, but about half of the patients answered that they had taken treatment for back problems the last year. The planned early weaning group had better results for self-image and satisfaction with treatment, but differences were small and of uncertain clinical importance (4).

**Table 3:** Sociodemographic characteristics in 381 Boston braced patients with late-onset juvenile or adolescent idiopathic scoliosis at mean follow-up of 23.4 years

Characteristic	A. Unplanned early weaning	B. Planned early weaning	C. Ordinary bracing
	*n* = 32	*n* = 59	*n* = 290
Educational level			
Primary school (9 year)	7	9	9
High school (12 year)	32	19	22
University college	61	72	69
Work status			
Working full time	78	75	78
Working part-time	4	7	7
Student/homemaker	6	7	5
On sick leave	0	3	2
Disability pension	12	8	8
Changed job because of back pain or disability	36	34	24
Scoliosis influenced my choice of education and job	34	28	29
Comorbidity	23	30	33
Smoking	34	13	20
Any treatment last year	52	59	56
Physiotherapy last year	19	35	27
Born children (*n* =303)	90	90	85
Pain in pregnancy (*n* = 303)	46	49	53

Percentages are given

**Table 4:** Quality of life score results in 381 Boston braced patients with late-onset juvenile or adolescent idiopathic scoliosis at mean follow-up of 23.4 years

Outcome	A. Unplanned early weaning	B. Planned early weaning	C. Ordinary bracing
	*n* = 32	*n* = 59	*n* = 290
Global Back Question^a^			
Excellent	5	20	79
Good	18	23	131
Fair	7	14	65
Poor	2	2	14
General Function Score (0–100)	9.7 (14.1)	4.9 (7.8)	7.0 (11.9)
Oswestry Disability Index (0–100)	12.1 (13.5)	7.2 (9.9)	8.4 (11.6)
EQ – 5D (−0.5 to 1.0)	0.75 (0.3)	0.83 (0.2)	0.82 (0.2)
EQ – VAS (0–100)	72.8 (18.9)	79.1 (19.6)	78.1 (17.4)
SRS-22 (0–5)			
Pain	4.0 (0.8)	4.2 (0.8)	4.1 (0.8)
Physical function	3.8 (0.8)	4.1 (0.7)	4.0 (0.7)
Mental health	4.0 (0.6)	4.2 (0.7)	4.1 (0.6)
Self–image	3.5 (0.8)	4.0 (0.7)^b^	3.7 (0.7)
Satisfaction	3.4 (0.9)	4.0 (1.0)^b^	3.7 (1.0)

Numbers or means (standard deviations) are given

^a^One patient in Group C did not answer this question

^b^Different from A (*p* < 0.01) and C (*p* < 0.05)

## Discussion

The aim of the present study was to compare early planned brace weaning with ordinary bracing and unplanned early brace weaning. The benefit of planned early weaning in immature patients was the shortened bracing time and good clinical results. Patient satisfaction and curve size at long-term follow-up suggest that patient selection for the planned procedure was successful. Even with resumed treatment they saved in average 2 years of bracing. Only one patient was operated, while surgery was increased in the groups with ordinary weaning and unplanned early weaning.

The patients who had planned early weaning were younger at brace start, had smaller curves, and better curve flexibility and curve redressment in per cent. All these factors may have influenced outcome. Due to the young age and early start of brace treatment the total treatment time was longer in the planned early weaning patients compared to those with ordinary brace treatment, but still considerably reduced relative to the expected time until skeletal maturity. At brace weaning the average primary curve measured about 21°. The choice was therefore to continue bracing until maturity or to stop before maturity. Curve size and expected time in brace were strong arguments to stop bracing, but still the selected patient to receive early weaning was not mature and the choice of early weaning was against guidelines. In average bone age at stop bracing was 1.9 years lower compared with ordinary bracing.

The grouping of patients according to planned and unplanned bracing adds information to the previously published study [7] by reporting results in those with stable reduced or unchanged curves who had aborted brace wear as planned early weaning before skeletal maturity.

Quality of life was not reduced after bracing in a recently published trial on the effectiveness of bracing [2]. This suggests that the psychological burden of bracing is overestimated. Still patients would prefer not to use the brace if the risk of surgery and long-term quality of life are comparable.

The main limitation of the present study is lack of randomisation. In clinical studies in general and in this old clinical study in particular, stringent selection of patients is hard to accomplish. Even if the selection of patients for planned early weaning was not completely stringent, the majority (81 %) of the patients had a primary curve of 25° or less which is considered to be a threshold value for bracing.

## Conclusions

We conclude that the benefit of planned early weaning was the shortened bracing time. It is our opinion that the procedure can be recommended in selected patients, in the present study they constituted 15 % of the patients braced. Planned early weaning may be attempted if curve reduction is stable and the primary curve is 25° or less in patients with several years of expected bracing. The patients should be monitored carefully and regularly at 4-6 months intervals until maturity, and a new brace should be prepared if the curve increases significantly. Ideally, this recommendation should be based on the findings from a future multi-center randomised study comparing planned early weaning and ordinary bracing in selected patients.
